# Two-level modeling approach to identify the regulatory dynamics capturing drug response heterogeneity in single-cells

**DOI:** 10.1038/s41598-021-99943-0

**Published:** 2021-10-21

**Authors:** Madalena Chaves, Luis C. Gomes-Pereira, Jérémie Roux

**Affiliations:** 1 Université Côte d’Azur, Inria, INRAE, CNRS, Sorbonne Université, Biocore Team, Sophia Antipolis, France; 2 Université Côte d’Azur, CNRS UMR 7284, Inserm U 1081, Institut de Recherche sur le Cancer et le Vieillissement de Nice, Centre Antoine Lacassagne, 06107 Nice, France

**Keywords:** Control theory, Regulatory networks

## Abstract

Single-cell multimodal technologies reveal the scales of cellular heterogeneity impairing cancer treatment, yet cell response dynamics remain largely underused to decipher the mechanisms of drug resistance they take part in. As the phenotypic heterogeneity of a clonal cell population informs on the capacity of each single-cell to recapitulate the whole range of observed behaviors, we developed a modeling approach utilizing single-cell response data to identify regulatory reactions driving population heterogeneity in drug response. Dynamic data of hundreds of HeLa cells treated with TNF-related apoptosis-inducing ligand (TRAIL) were used to characterize the fate-determining kinetic parameters of an apoptosis receptor reaction model. Selected reactions sets were augmented to incorporate a mechanism that leads to the separation of the opposing response phenotypes. Using a positive feedback loop motif to identify the reaction set, we show that caspase-8 is able to encapsulate high levels of heterogeneity by introducing a response delay and amplifying the initial differences arising from natural protein expression variability. Our approach enables the identification of fate-determining reactions that drive the population response heterogeneity, providing regulatory targets to curb the cell dynamics of drug resistance.

## Introduction

Cell response heterogeneity is an inherent feature of clonal cell populations^1,2^, which results in the graded response observed after drug treatment, leaving drug-tolerant persiters behind in the treated cell population. In the case of cancer drugs, this incomplete therapeutic response of sensitive tumor clones is a first step prior to acquiring de novo mutations that enable stable drug resistance and subsequent therapeutic failure^3–5^. We and others have shown that drug-tolerant persisters can emerge from differences in cell response dynamics^6,7^. Cell-to-cell variability in these response dynamics actually impairs drug efficacy, evidenced at all drugs IC_50_^8,9^.

Single-cell multimodal technologies have exposed the extent of heterogeneity allowing isogenic cells to evade cancer drug treatments, revealing cellular heterogeneity as a emergent feature to characterize and predict cellular states^10^, anticipate cellular behavior^9^, and possibly design cancer treatment strategies^11^. Yet single-cell dynamics obtained from live-cell assays remain only partially exploited in modeling approaches, to investigate the role of cellular heterogeneity in drug response. Recent experimental studies have suggested that cell-to-cell heterogeneity has a functional role in cell decision after treatment^6,7^, that can regulate phenotypic changes at the population level, as it was further shown computationally^12^. The latter live-cell experiments have revealed that this graded therapeutic response (fractional killing) is in large part the results of cell-to-cell differences in drug response dynamics, for which mathematical models -in the form of ordinary differential equations (ODE), are particularly well suited to decipher.

The extrinsic apoptosis pathway triggered by death receptor agonists, such as tumor-necrosis-factor-related apoptosis-inducing ligand (TRAIL) and Fas ligand, are signaling pathways of choice for modeling drug response heterogeneity, as the results of each cell decision is binary, with an important impact on the overall cancer drug treatment efficacy^13–16^. In this signaling pathway, TRAIL binds to its receptors (TRAIL receptor 1/2, TRAILR1/2 or DR4/DR5) as a trimer, and enables the formation of a protein complex, the death-inducing signaling complex (DISC), including Fas-associated protein with death domain (FADD), pro-caspase-8 and pro-caspase-10 and cellular FLICE-like inhibitory protein (cFLIP), as well as other regulatory proteins which stoichiometry or arrangement within the complex (such as initiation of caspase filaments) is highly regulated^17–21^. Solely with these main regulatory proteins, isoforms and homologs provide additional levels of regulations. While short isoforms of FLIP (FLIP-S and -R) may simply inhibit caspase-8 activity, the long isoform FLIP-L can have differential impacts on signaling, depending on its concentration at the DISC^22,23^. Caspase-8 homolog, Caspase-10, has been shown also to favor DISC signaling toward pro-survival phenotypes^24^. Although in some cell types, proper caspase-8 activation can directly balance the effector caspase inhibitor XIAP and yield to cell death, more commonly, apoptosis execution will require caspase-8 dependent cleavage of Bid, a BH3-only protein, which will induce the formation of Bak/Bax pores in the outer mitochondrial membrane to induce sudden activation of caspase-3 causing cell death^25,26^. Although the mitochondrial outer membrane permeabilization (MOMP) is typically considered as the “point of no return” in the apoptosis pathway, there is a large body of evidence describing the relationship between apoptosis execution and post-MOMP events^15,26–29^. In^28^ for example, cell response heterogeneity is linked to apoptosis impairment subsequent to MOMP, while in^27^, enhanced glucose utilization is suggested to regulate apoptosis post-MOMP in cancer cells. Here we have focused the apoptotic network on receptors reactions as we have previously shown that a caspase-8 activity threshold determined cell fate^7^.

The current models of this pathway as well as early mathematical models of apoptosis^30,31^, described the activation of a (pro-apoptotic) cascade of caspases, and its interaction with an anti-apoptotic pathway which involves the activation of an inhibitor of apoptosis proteins (XIAP) itself regulated by Nuclear Factor $\kappa$B.

In particular,^31^ first introduced the idea of a possible feedback loop between caspase 8 and caspase 3, which allowed recovering a bi-modal cell response (cell death or survival) due to differences in initial concentrations of the proteins, such as XIAP, with the existence of a separatrix dividing the phase plane into regions corresponding either to these opposing phenotypes^32^. This hypothesis of a feedback between initiator and executioner caspases has been further used and explored in several models, although experimental evidence for such type of feedback is still not conclusive^33^.

Other works have aimed at explaining the high level of heterogeneity observed in cells treated with cancer drugs^34^. To do so, the authors had added a genetic layer to a comprehensive model of apoptosis^15^, by introducing an ON/OFF promoter activity model, to study the effect of stochastic protein synthesis in generating a large variety of cell responses. More recently, the idea of a separatrix was further developed to suggest that a saddle node can introduce the mechanism for cell decision between death or survival, with each phenotype corresponding to a different steady state, while perturbations in initial conditions impact fate probabilities^35^.

Although cell death models have provided a global view of the apoptosis pathways and reproduce some of its heterogeneous characteristics, they have not been calibrated to time-resolved experimental trajectories of single cells, limiting the models interpretation for its dynamical properties and confounding the mechanisms at the origin of response heterogeneity. With few exceptions, only averaged observations are typically used in simulating heterogeneous cell dynamics, but mean cell response rarely equals any of the single-cell’s behavior of the population and it never captures outliers features that are often at the disease origins^36^. Since the heterogeneity observed in a clonal cell population contains the information on each cell’s capacity to elicit the range of response behaviors, we propose here an approach to discriminate the reaction sets driving population heterogeneity in order to identify new regulatory reactions (motifs), starting from an elementary network of reactions. Using live-cell microscopy, we have previously shown that the apoptotic response of each single cell is determined by the activation rate of caspase-8 (hereafter C8)^7^, revealing a wide range of response profiles within the isogenic cell population, without further genetic induction nor race with other signaling pathways. In addition, some outliers were shown to exhibit a commitment to cell death very early after drug treatment^9^, suggesting a sufficient number of reactions at the receptor level to generate diverse responses and opposing fates. In the present study, we therefore developed a two-level approach in which a minimal apoptosis receptor reaction model (ARRM) is first calibrated to experimental single-cell trajectories from a clonal population of cells under therapeutic treatment, in order to characterize the differential dynamics between the drug response phenotypes. With that, we obtained a selected group of reactions that have been used in a second level, to augment the model structure with new regulatory mechanisms that could recapitulate a phenotype separation, as we found, with only the natural heterogeneity observed in cell populations.

## Results

The cell-to-cell variability in signaling dynamics is attributed in large part to natural fluctuations in protein expression^32,34,37^, which extent may vary between proteins (from 20 to 30% with 2–3 days of mixing time^38–40^). Since experimental studies of single-cell response to cytotoxic drugs pertain to clonal cells^7^, both response phenotypes (drug sensitive and resistant) should emerge from the same pathway structure (sister cells), with variability originating from fluctuations in protein initial concentrations. In principle, a single model calibrated on a prototypical cell could serve to reproduce the population heterogeneity with an array of simulations (varying initial conditions only), once the model sufficiently describes the regulatory processes involved in the cell response. To test this hypothesis we developed a modeling approach starting with a simple mathematical model and incrementing additional reactions as needed to obtain the minimal structure that encapsulates the population behavior in one model topology.

### Two-level modeling approach

**Figure 1:**
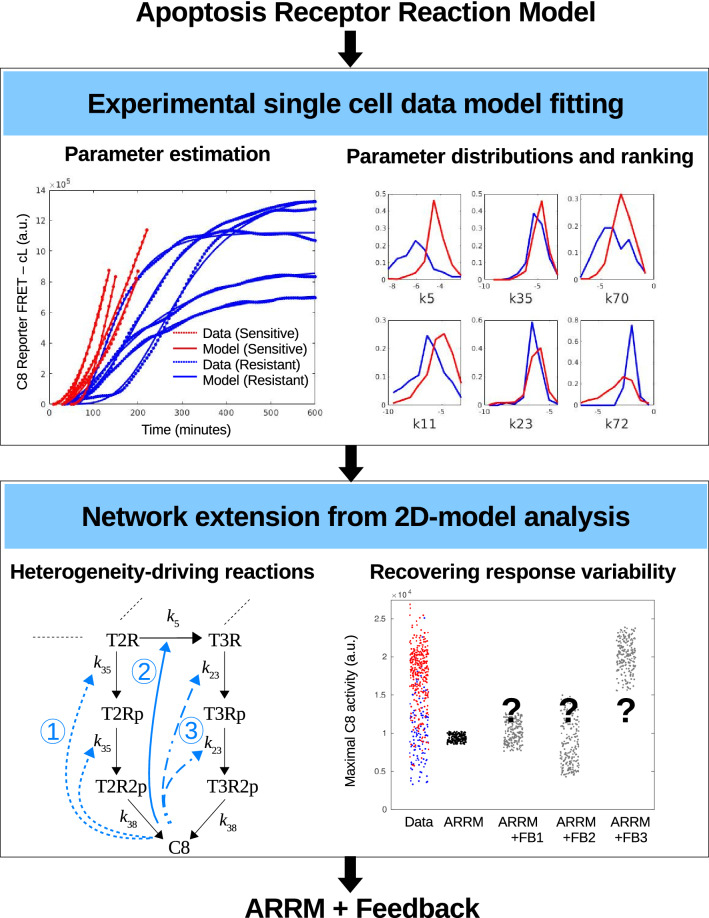
The two-level approach from model development and calibration to network analysis and heterogeneity prediction. Top row: An apoptosis receptor reaction model (ARRM) is first constructed and fitted to experimental single cell trajectories, to obtain parameter sets for every sensitive (red curves) and resistant (blue curves) cells. Comparison and ranking of parameter distributions indicates a small subset of reactions (five out of 32) distinguishing between drug response phenotypes. Bottom row: Since ARRM is not sufficient to explain the variability observed in experiments in response to 20% variation in initial conditions (measured by the maximal slope of C8 Reporter FRET signal, a proxy for maximal C8 activity^7^), we analyse the signaling network with extra regulatory processes, in the form of a positive feedback loop from one of the main proteins to a key heterogeneity-driving reaction identified previously. Theoretical analysis of a 2-dimensional (2D) model, predicts the network configurations which are capable of generating large heterogeneity as a function initial conditions only. Each set of light blue arrows (dashed, solid, or dash-dotted) forms a possible configuration of the extended ARRM+Feedback model. The new feedback configurations are also fitted to single cell data and tested for their capacity to generate large response variability.

In brief, we first build the mathematical model of the signaling pathway of interest, here describing the death receptors reactions that initiate extrinsic apoptosis pathway (apoptosis receptor reaction model, hereafter ARRM). This model represents a single model topology for all sister cells. (Further genomic induction of survival pathways is not considered here, as sister cells engaged their decision as soon as 50 min after drug addition^9^.) The first step in our analysis is based on model fitting to single-cell data^7^, to obtain one parameter set for each cell, and the subsequent analysis of the parameters. To characterize the main cellular properties distinguishing between the two phenotypes, we compare the parameter distributions obtained from fitting sensitive and resistant cells (see Fig. 1, top), highlighting the subset of parameters (here a group of 5 out of 32 parameters) that exhibit most of the heterogeneity observed.

In the second step we further analyze the identified reactions, by extending the apoptosis pathway with one extra regulatory reaction to a reaction of the set. With the help of a compact model of two variables we next validate the chosen regulatory process; here a positive feedback loop from the C8 variable (see Fig. 1, bottom). We then validate, with a fixed set of parameters, that the model extended with a new regulatory reaction, properly recapitulates the population heterogeneity with natural protein variability and exhibits a separation between drug response phenotypes. The approach also enables further understanding of the regulatory dynamics, presented next.

### Apoptosis receptor reaction model (ARRM)

**Figure 2:**
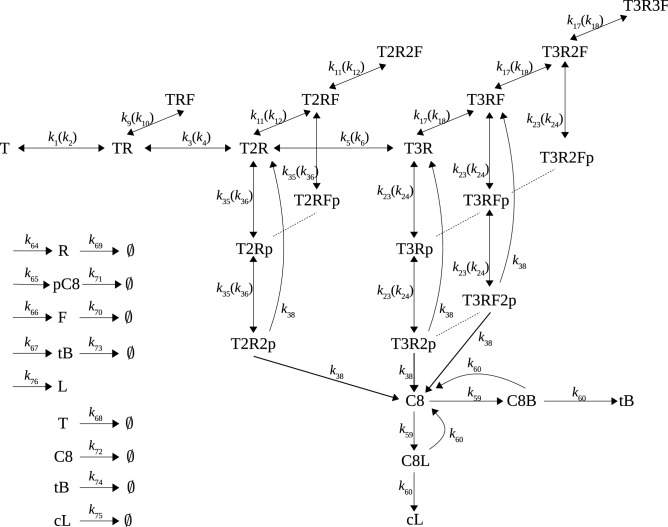
A schematic representation of the Apoptosis Receptor Reaction Model (ARRM). Binding of TRAIL (*T*) successively to one, two or three receptors (resp., *TR*, *T*2*R*, *T*3*R*), with possible binding to one or more molecules of FLIP (resp., *TRF*, *T*2*RF*, *T*2*R*2*F*, etc.), and to one or two molecules of pro-caspase 8 (resp., *T*2*Rp*, *T*2*R*2*p*, *T*3*Rp*, *T*3*R*2*p*). Any combination of three molecules of either FLIP or pro-caspase 8 can bind to *T*3*R*. *L* represents the synthetic Fluorescent Probe (IC-RP^7^), which is cleaved by C8 in competition with its natural substrate Bid. The measured quantity is thus *cL*. In addition to the binding, dissociation, and activation reactions, the model also considers synthesis and degradation ($\emptyset$) rates for its proteins, as indicated at left. Forward and backward reactions are given outside and inside parentheses respectively.

ARRM is a system of ordinary differential equations whose kinetics are exclusively given by the laws of mass-action, it expands the receptor module of previously published models of extrinsic apoptosis^26,32^ (see Fig. 2). The proteins described in ARRM are: the death-receptor ligand TRAIL, a trimeric ligand that can interact with three receptor molecules; the death receptor (R) at the plasma membrane; pro-caspase 8 (pC8); FLIP, a protein which competes with pC8 to bind the membrane receptor and thus plays the role of an inhibitor of caspase 8 activation; C8; Bid; the fluorescent reporter for C8 activity (L); and the cleaved fluorescent reporter (cL), the equivalent to the FRET signal measured in the corresponding live-cell experiments^7^.

As illustrated in Fig. 2, the model is driven by the successive binding of TRAIL to three death receptors at the cell membrane, forming the complexes TRAIL:R, TRAIL:2R, and TRAIL:3R. From these central complexes, the system branches into two alternatives, either binding of each receptor to pro-caspase 8 or to the anti-apoptotic protein FLIP. These lead to different combinations of the TRAIL-receptor complexes with one, two or three molecules of pC8 and/or FLIP:

$$\begin{aligned} \text{ TRAIL:}{\it nR:ipC8:}j\text{ FLIP },\ \ n,j\in \{0,1,2,3\},\ i\in \{0,1,2\}, \end{aligned}$$

where pC8 and FLIP molecules can only bind if at least one receptor is bound to TRAIL ($i\le n$, $j\le n$), and each receptor can allocate one molecule ($i+j\le 3$). In addition, pC8 can bind only TRAIL-receptor complexes with two or more receptors (that is $n=1$ implies $i=0$).

Active C8 is produced from intermediate complexes with two pC8 molecules, meaning that TRAIL:2R:2pC8, TRAIL:3R:2pC8, and TRAIL:3R:2pC8:FLIP are the three complexes contributing to cell signaling. Therefore, no complexes with more than two pC8 molecules can be formed (that is $i\le 2$). In the case of the trimeric complexes, we do not consider the relative insertion/position/distance between the two pC8 molecules in the attached receptors to affect C8 activation dynamics. The last reactions in the model represent the cleavage of both C8 substrates, Bid and L (the fluorescent reporter). The cleaved product of the fluorescent reporter, cL, corresponds to the experimental time-resolved signal, a measure of C8 activity dynamics^7^.

A table listing all the reactions and corresponding parameters is given in Supplementary Table 1. Detailed mass-action models typically involve many parameters, most of which are unknown. To overcome this problem, we first reduce the number of distinct parameter by hypothesizing that some reactions share the same rates: for instance, the binding of pC8 to a receptor depends on the number of receptors already bound to TRAIL, but is independent of the amount of receptors already occupied (see rates labeled $k_{24}$ and $k_{35}$). Likewise, for the binding of FLIP to a receptor (see rates labeled $k_{9}$, $k_{11}$, and $k_{17}$). Similarly, the dissociation rates of one pC8 or one FLIP molecule depend on the number of receptors bound to TRAIL (see $k_{10}$, $k_{12}$, $k_{18}$ for FLIP, and $k_{24}$, $k_{36}$ for pC8). The same C8 activation rates ($k_{38}$) are assumed for each of the three complexes.

These simplifications provided a model with 28 variables and a total of 32 parameters.

### Model calibration with experimental single-cell data

Our dataset comprises three groups of single-cell measurements of C8 activation, each consisting of about 400 live cells exposed to 25, 50, or 100 ng/ml of TRAIL and tracked for 10 h, with images captured every 5 min (single-cell trajectory dataset publicly available^7^). Here, we use the 50 ng/ml group as a training set to our model, and validate it with the other two groups. The dataset has the following three remarkable characteristics: first, a deterministic aspect in the response, consisting of a sigmoidal-type increase in FRET signal, followed by a saturation region; second, a stochastic element reflected in the large variability in C8 activation rate between cells, or time to reach the saturation region. The third element is the physiological response, which divides the entire group of cells into two sub-populations of either surviving or sensitive cells (the latter respond to the drug by committing to cell death well before the 10 h). Among the sensitive cells, the hour of cell death also admits a large variability. The sensitive and resistant phenotypes are clearly differentiated within the first hours of tracking. To quantify the differences between single-cell curves Roux *et al.* compared the maximal C8 activity of each cell (defined as the maximal time derivative of the C8 reporter FRET signal), and they observed that all sensitive cells have a higher maximal C8 activity^7^. Here, we refer to the maximal C8 activity as the ***maximal slope***, as we calibrate our model to the C8 reporter FRET signal (cL in the model).

The calibration of ARRM to single-cell dataset is aimed at characterizing the main cellular properties distinguishing the two phenotypes within a population of clonal cells. ARRM represents the main deterministic steps in C8 activation reactions: by separately fitting each single cell trajectory to the model, we intent to obtain one specific parameter set for each cell, characterizing its dynamic response. The aforementioned stochastic elements is to be captured in the parameter distributions within each sub-population and we expect larger differences between physiological responses to appear from comparison of parameter distributions over the two sub-populations.

All model parameters, including synthesis and degradation rates, and rate constants were fitted to the data, for each cell separately, as described in “Methods”, to obtain one set of parameters for each cell. Some examples are shown in Fig. 1 (top left panel) as well as Supplementary Figs. 2 and 3. To test the capacity of ARRM to reproduce the experimental heterogeneity, we computed the variability in cell response generated by a coefficient of variability (CV) of 20% in initial conditions. For a prototypical resistant cell, we show here the range of maximal slopes is not reproduced by ARRM by varying only the initial amounts of baseline proteins (Fig. 1, bottom right panel). In either case, the delay in onset of C8 activation is not captured, suggesting that this property is not dependent on the baseline amounts of proteins. Altogether with previous work evaluating the effect stochastic fluctuations in apoptosis receptor reactions^41^, these simulations indicate that ARRM is not sufficient to explain the differences observed in experimental trajectories of C8 activation dynamics.

### Parameter distributions analysis and ranking

Since both resistant and sensitive cells originate from the same clonal population, the parameters should theoretically be the same for both response phenotypes. However as we show, variability in initial protein concentrations is not sufficient to separate the two phenotypes, suggesting that additional reactions should be considered in the model. We hypothesize that kinetic parameter’s flexibility might inform on the deficiency of a regulatory step in the model structure. The analysis of the distributions of parameter values over each phenotype should reflect the differences in the dynamical evolution that can predict the sub-population response to the drug. The next series of analyses are aimed at identifying the heterogeneity-driving reactions that would require an extra regulatory step in order to better separate the two opposing response and recapitulate the differences observed in experimental trajectories of C8 activation.

**Figure 3:**
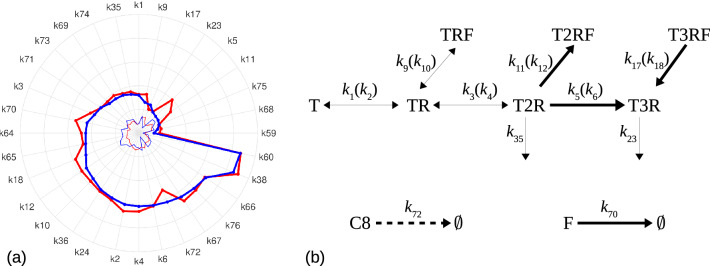
Parameter ranking. (**a**) Comparison of the average values of logarithms of parameters, for sensitive (red) and resistant (blue) cells. Parameters are ordered increasingly on resistant cells values. (**b**) Partial ARRM scheme showing the main differences between the two phenotypes: reactions enhanced (bold arrows) or diminished (dashed arrow) in the sensitive phenotype.

To perform these analyses, the parameters were first labeled and scaled (as indicated in “Methods” section). The normalized distributions for six representative parameters are shown in Fig. 1 (top right panel). The average parameters for both phenotypes are illustrated in Fig. 3. Interestingly the two sub-populations share globally the same parameter trends but a distinguishing pattern emerges: the forward binding rates $k_5$ and $k_{11}$, and the dissociation rate $k_{18}$ are all larger for the sensitive phenotype. A significant difference (two orders of magnitude) is observed for $k_{5}$, which characterizes the receptor trimerization of TRAIL. Combined with $k_{18}$, which describes the dissociation of FLIP with relapse to the trimeric receptor-TRAIL complex, these parameter differences suggest that sensitive cells tend to rapidly build their trimer complex for a faster increase of caspase-8 activation. This interpretation is further strengthened by a higher FLIP degradation rate ($k_{70}$) in sensitive cells, a factor contributing to more availability of non sequestered receptor-TRAIL complexes.

In addition, C8 degradation rate $k_{72}$ is higher in resistant cells which allows another clear distinction between the two phenotypes, since this parameter is the most accurately estimated (lowest standard deviation), indicating its finely tuned property. Together, these results point to the activation dynamics of receptor dimers or trimers as the main difference between sensitive and resistant phenotypes.

### Network extension from candidate parameters

Our parameter analysis highlighted a group of only five out of 32 parameters representing the reactions that more strongly differ between the two phenotypes. It also appeared that this group of reactions forms an enhanced pathway, favored by sensitive cells, which suggests the existence of a fine-tuning regulatory mechanism allowing the cell to better adjust its activities. In the next steps we therefore sought a mechanism that allows the cell to better fine-tune its responses to initial amounts of molecules, starting from a common topology. (In modeling terms, this means using the same model with fixed parameters, but with different initial conditions leading to different phenotypes.) As stated above we hypothesized here, that one or more of the selected reactions have an extra regulation step (or represent an aggregate of missing reactions), not described in original ARRM. To test this hypothesis in a systematic way, but without adding extra unknown variables to the model, we extended the network (Fig. 2) by allowing a given reaction ($k_r$) to become dependent on one variable *X* of the model, in the form of a feedback loop:

1 $$\begin{aligned} K_r(X)~=~k_r\frac{X^2}{X^2+k_{fbk}^2}. \end{aligned}$$

A positive feedback loop is a common mechanism used by biological systems for decision making (cell differentiation, cell development^42,43^), since it may generate the existence of two stable steady states with distinct protein concentrations, representing two different cell states or cellular responses. This mechanism also helps to amplify the differences between initial conditions. The role of the reaction $k_r$ becomes twofold:

(i) if *X* remains sufficiently high above the threshold $k_{fbk}$, then the parameter $k_r$ remains approximately unchanged ($K_r\approx k_r$);
(ii) conversely, low concentrations of *X* ($\ll k_{fbk}$) imply a significant variation of $k_r$ and $K_r< k_r$, hence a lower activity rate for reaction *r*.

The function $K_r$ represents an “effective” feedback loop, which is considered here as a tool to describe a potential additional regulation rather than a direct “biological” feedback loop.

We will use this effective feedback loop to systematically study new model architectures, now referred to as ARRM+Feedback. We next tested whether these new model architectures could reproduce the heterogeneity in cell response and which parameters should be treated as dynamically varying (outstanding candidates being the ones that most significantly reflect the differences between the two phenotypes shown previously, Fig. 3).

### A 2D-model analysis to investigate the role of feedback in heterogeneity

To study the mechanisms at play in ARRM+Feedback, we first used a basic surrogate model involving one receptor-ligand binding followed by complex $\textit{T:R}$ formation and subsequent activation of the target protein *C*8, as described by the reactions $T+R\leftrightharpoons \textit{T:R}\rightarrow C8+\textit{T:R}$. In addition, we assumed conservation of mass for the total (free or bound) receptors and TRAIL, respectively given by $R_0=R(t)+\textit{T:R}(t)$ and $T_0=T(t)+\textit{T:R}(t)$. These conservation laws allowed us to simplify the model and obtain a system of two differential equations:

2 $$\begin{aligned} \frac{d\textit{T:R}}{dt}= & {} k_{on} (R_0-\textit{T:R}) (T_0-\textit{T:R}) -k_{off}\,\textit{T:R}\end{aligned}$$

3 $$\begin{aligned} \frac{dC8}{dt}= & {} k_{act}\, \textit{T:R}- k_{deg} C8. \end{aligned}$$

The four parameters ($k_{on}$,$k_{off}$,$k_{act}$, and $k_{deg}$) can be modified as in (1) to depend on one of the variables *C*8 or $\textit{T:R}$, each combination corresponding to a different feedback loop. Based on the parameter distributions obtained for ARRM, we compared the conformations induced by a feedback loop on $k_{on}$,$k_{act}$, or $k_{deg}$, from the variables *C*8, $R\equiv R_0-\textit{T:R}$, and $1/C8\approx FLIP$. (FLIP is a catalytically inactive caspase-8/-10 homologue, which competes with caspase-8 for receptor binding; although its short isoform acts as an inhibitor only, its long isoform can also increase C8 activation rates in some specific contexts that were simplified here^22^).

To understand the dynamics of the system under the different feedback loops we analyzed the nullclines (i.e. the points satisfying $d\textit{T:R}/dt=0$ and $dC8/dt=0$). More specifically, we studied the changes induced in the form of the nullclines by each of the feedback loops, as described in “Methods” section.

**Figure 4:**
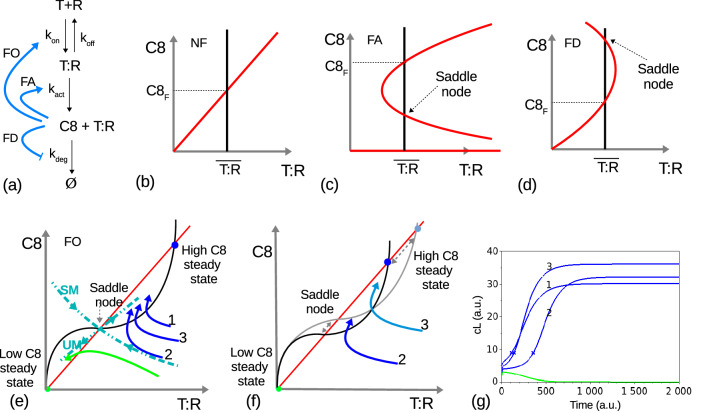
The nullclines and fixed points of the 2D system (2–3), for different network configurations summarized in (**a**): (**b**) in the absence of feedback loops, NF; (**c**) with feedback loop from *C*8 to $k_{act}$, FA; (**d**) with feedback loop from *C*8 to $k_{deg}$, FD; (**e**) with feedback loop from *C*8 to $k_{on}$, FO. The three feedback loops are *positive* since the final effect of C8 is to increase its own concentration. A schematic view of the 2D system phase plane around the saddle node is shown in case (**e**): trajectories first approach the node along the stable manifold (dashed curve SM) and then are repelled from the node along the unstable manifold (dashed curve UM). Depending on its initial starting point, each trajectory makes a “decision” to converge towards the high or low C8 steady states (respectively, blue and green trajectories); (**f**) Variation in initial conditions also induces changes in the conserved quantities of the system: in this case $R_0$ changes the total amount of receptors and affects the value of steady states, as represented by the black and grey nullclines; (**g**) A large variability is obtained from a small neighborhood of initial conditions, both in terms of maximal slope and activation delay.

We found the original network and three possible feedback configurations, all leading to the appearance of new intersection points between the red and black nullclines and creating a saddle node (Fig. 4). A saddle node is an unstable steady state which defines the system dynamics as follows: trajectories approach the saddle node along one direction (the stable manifold, SM) and recede from it along an orthogonal direction (the unstable manifold, UM). Thus, trajectories starting on one side of the saddle node will first converge towards it (see Fig. 4e, trajectories 1, 2 and 3), until they cross the $\textit{T:R}$-nullcline (Fig. 4, black line); here, trajectories switch their direction to converge towards one of the high or low C8 steady states. The low steady state represents cells that do not respond, while the high one corresponds to both phenotypes, as discussed next.

These dynamics introduce two new features: first, a large variability in cell responses is obtained from a small neighborhood of initial conditions, based on $R_0$ and $C8_0$ (see Fig. 4e–g); second, trajectories starting from neighboring initial conditions at time $t=0$ may reach the $\textit{T:R}$-nullcline at very different times: in fact, the feedback configuration introduces a wide range of time delays for neighbor trajectories (see Fig. 4g). A longer delay capacity is restricted to the trajectories which are closer to the stable manifold of the saddle node. Importantly, these observations do not imply the existence of a well defined separatrix dividing the space into initial conditions leading to cell death or survival, as suggested earlier in^32^ or more recently in^35^. Although a saddle node introduces a division between two locally stable steady states in our model, we found that both resistant and sensitive phenotypes correspond to the same high C8 steady state, while the low steady state corresponds to non responding cells. Therefore, the role of the saddle node is to augment the differences between initial concentrations, so as to obtain a high degree of variability in the various response properties such as response delay, maximal slope, or final state, providing a basis for heterogeneity in cell response (Fig. 4e–g).

### Identification of heterogeneity-driving reactions

To test whether the new model topology ARRM+Feedback had indeed the capacity to reproduce the heterogeneity in cell response, we designed a process to systematically evaluate the role of each reaction in the cell death decision, by modifying the model according to Eq. (1). Based on the parameter distribution results, we selected two groups of reactions: $\mathcal {K}_{dist}=\{k_5,k_{11},k_{18},k_{70},k_{72}\}$ to represent the differences detected in Fig. 3 and $\mathcal {K}_{act}=\{k_{23},k_{35}\}$ to reflect the analog of parameter $k_{act}$ in the 2D model. The basic proteins involved in ARRM—caspase-8, pro-caspase-C8, receptors and FLIP—are in turn considered as the feedback variable *X*, giving rise to feedback loops of the form $K_r(X)$ with $r\in \{5,11,18,23,35,70,72\}$ and $X\in \{C8,pC8,R,F\}$. Each of these feedback loops was successively introduced in ARRM to evaluate the capacity for heterogeneity to cell response (the process is further described in “Methods” section.)

The output of the process consisted only of the cells that have the capacity to generate high variability of responses from small differences in the initial amounts of proteins (20% cv), and therefore correspond to a *universal profile*. All the cells with a universal profile (Supplementary Fig. 1) were in the resistant sub-population, with a fairly high final state and an average maximal slope. In this way, the corresponding model could generate both the very sensitive and the very resistant cells (Fig. 6a). In cells with such profiles, response heterogeneity was indeed determined solely by initial proteins variations.

In summary, analyses of the ARRM+Feedback (Table 1) revealed several key findings: first, only very few feedback configurations effectively have the capacity to generate high heterogeneity in cell responses. It is interesting to observe that all of these require active C8 as a feedback variable, while feedback from pro-caspase 8 does not generate heterogeneity. All universal profiles correspond to configurations predicted by the 2D model, that is, those where a saddle node exists to play the role of initial conditions amplifier. Second, feedback through C8 activation parameters $k_{23}$ and $k_{35}$, had the higher capacity for heterogeneity, perhaps because these parameters are linked to C8 expression by other (shorter) pathways. C8 feedbacks through parameters $k_5$ or $k_{72}$, which strongly distinguished between phenotypes, also exhibited high heterogeneity, confirming our hypotheses.

**Table 1:** Cell profiles with high heterogeneity capacity, for selected feedback reactions and variables in ARRM.

Feedback reaction, $k_r$	Feedback variable, *X*			
	C8	pC8	R	F
Correspondence [2D]	[C8]	[–]	[R0-T:R]	[1/C8]
***k***_***5***_ **[*k***_***on***_] **formation of T:2R**	**6**	0	0	2
$k_{11}$ [–] bind F to T:2R:*	1	0	0	0
$k_{18}$ [–] unbind F from T:3R:*	0	0	0	0
$k_{70}$ [–] degradation FLIP	0	0	0	0
***k***_***72***_ **[*k***_***deg***_**] degradation caspase-8**	**6**	0	0	0
***k***_***23***_ **[*k***_***act***_] bind pC8 to T:3R:*	**16**	0	0	0
***k***_***35***_** [*k***_***act***_] bind pC8 to T:2R:*	**15**	0	0	0

The equivalent reactions and variables in the theoretical 2D model are shown between brackets. The reaction parameters in bold correspond to the configurations for which the 2D model predicts universal profiles.

### Initial conditions discriminate phenotypes in extended model

Finally, in the last step of the approach, we now fixed the model topology and parameter set to one of the universal cell profiles and estimated only the initial conditions of the main four proteins (Receptors, FLIP, pro-caspase 8, and Bid) for each experimental cell trajectory.

**Figure 5:**
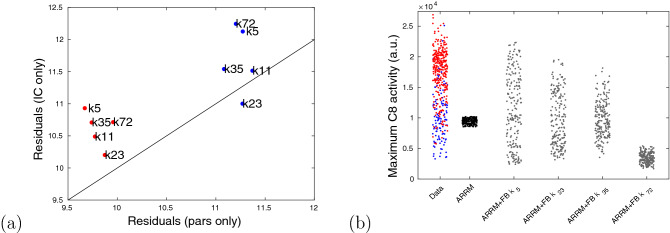
Comparison of different ARRM+Feedback configurations. (**a**) Average residuals for the feedbacks, $k_i(C8)$. Each configuration was fitted to all 414 cells, first estimating all parameters (“pars” only, *x*-axis) and then fixing the parameters at those of a universal cell and estimating all initial conditions (IC only, *y*-axis). Red and blue dots represent average residuals among sensitive and resistant phenotypes, respectively. The straight line represents the points $y=x$; (**b**) The maximal C8 activity (maximal slope of C8 Reporter FRET signal) generated by each configuration, in response to 20% variation in initial conditions (200 simulated cells randomly generated for each model).

To measure the goodness of fit of each configuration, we first compared average residuals from parameters-only to initial conditions-only estimates. We observed that the ARRM+Feedback topology $k_{23}(C8)$ had the best performance, with a decrease in the residuals by an initial conditions-only estimation for the resistant cells (Fig. 5). In other words, the topology $k_{23}(C8)$ not only reproduces high heterogeneity but also improves the model fits by calibrating initial conditions only. This striking outcome further valued the approach, since this last calibration relies on estimating only 4 unknowns, hence much fewer degrees of freedom than in the case of the parameters-only calibration with 32 unknowns.

**Figure 6:**
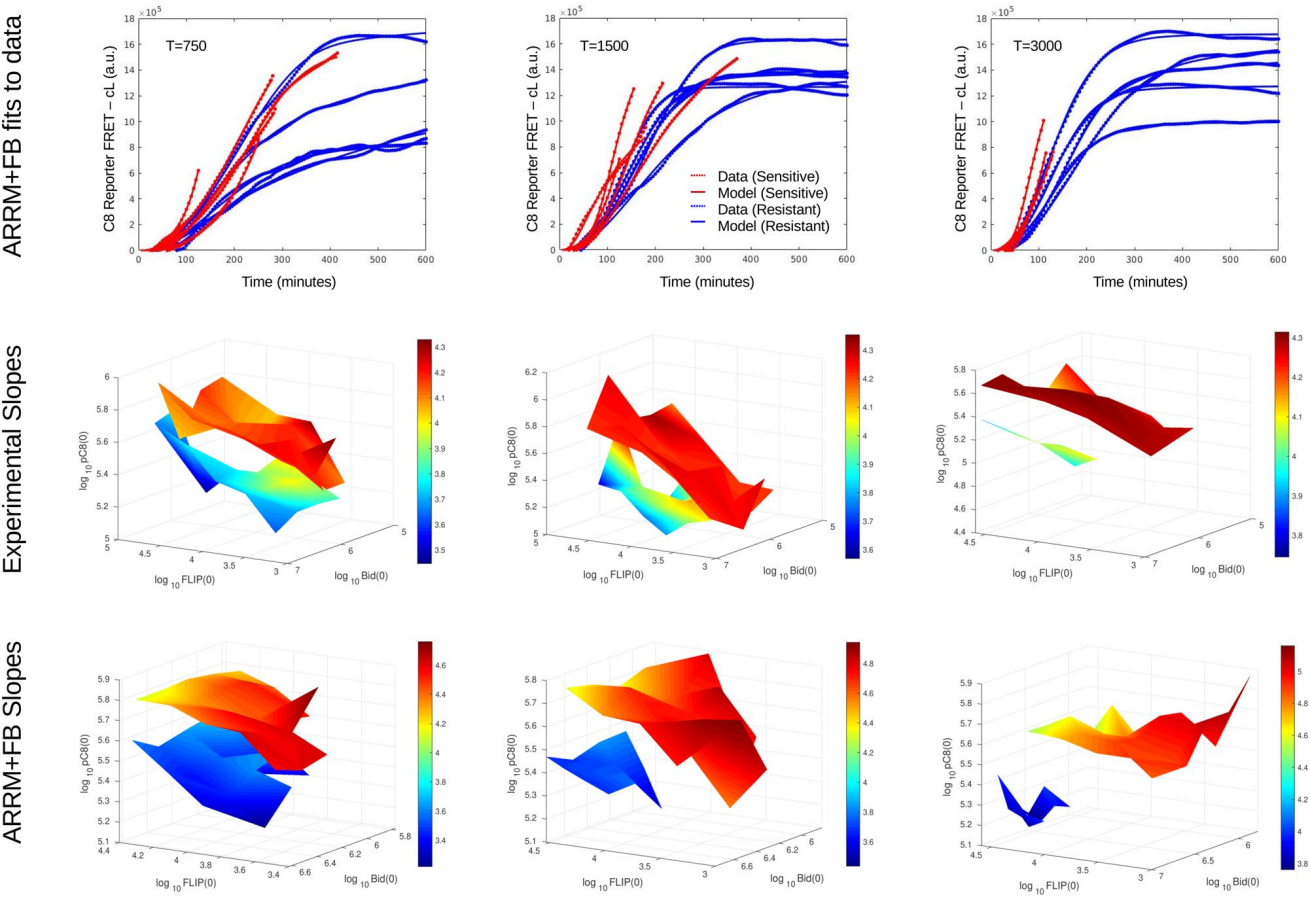
Estimating initial conditions for all cells with ARRM+Feedback $k_{23}(C8)$ for the three TRAIL dose groups, $T=25\; \text{ng}/\text{ml} \approx 750$ molecules, $T=50\;\text{ng}/\text{ml}\approx 1500$, and $T=100\; \text{ng}/\text{ml}\approx 3000$ molecules. In the middle and bottom rows, base 10 logarithms of all quantities are used. Top row: Examples of fits for resistant (blue) and sensitive (red) cells. Middle row: Projection of the estimated experimental conditions into the (FLIP,Bid,pC8) space. Each surface corresponds to one phenotype (blue tones: resistant; red tones: sensitive). To obtain each surface, we averaged the values of pC8(0) over intervals of (FLIP,Bid). The surfaces are colored according to maximal slope values, also averaged over intervals of (FLIP,Bid). Bottom row: Comparison to model results. Random initial conditions (about 400) were generated for each TRAIL concentration and the maximal slopes were measured. The experimental maximal slope-thresholds separating resistant and sensitive cells were used to obtain two phenotypes. The model results are analyzed in the same way as the experimental results in the middle row.

To further validate ARRM+Feedback $k_{23}(C8)$, we next used this configuration to estimate the four initial conditions in the remaining data groups, with 25 and 100 ng/ml corresponding to $T(0)=750$ and 3000, respectively. Remarkably, all cells were accurately fitted (Fig. 6, top row) and the initial conditions distribution across the maximal slopes was consistently similar for all groups. In addition, two clear features distinguishing between phenotypes stood out: resistant cells (blue tones) always had lower amounts of pro-caspase 8 and, as TRAIL dose increases, the resistant cells surface with lower maximal slopes seemed to be directly linked to higher amounts of Bid (Fig. 6, middle row).

The distribution of experimental initial conditions was also compared with model-generated results (Fig. 6, bottom row). For each TRAIL dose, a set of about 400 initial conditions were randomly generated out of a uniform distribution and, for each corresponding curve, the maximal slopes were measured. To obtain a separation between phenotypes, we used the maximal slope-thresholds (as computed in^7^) for each TRAIL dose. We next performed the visualization on the three-dimensional space (FLIP, Bid, pC8), with the maximal slope (color-coded, Fig. 6). As shown, we observed a remarkable qualitative equivalence between the experimental and model 3D surfaces. The model reproduced the differences in pC8(0) between resistant and sensitive surfaces as TRAIL increases, and the fraction of resistant cells (approximate size of the blue surfaces) were very similar. The general trends were also the same in both cases, namely higher maximal slopes corresponding to sensitive cells which had a high pC8(0) and low FLIP(0). These results strongly support the presence of a feedback of the form $k_{23}(C8)$ in the extrinsic apoptosis pathway and substantiate the hypothesis that protein expression heterogeneity (initial conditions noise) is largely accountable for cell decision in response to treatment.

## Discussion and conclusions

The advent of single-cell technologies using live-cell microscopy, microfluidics and computational approaches has allowed the study of cell-to-cell dynamic variability^6,7,9,44^ to illustrate its role in non-genetic drug resistance. From these seminal studies, several fundamental observations laid the basis of the approach presented in this work. First, cell fate decision can occur very early after drug treatment, and in the case of the apoptotic pathway, right upon caspase-8 activation following death-receptor ligand binding. Second, there is a high level of variability in single-cell response among a clonal population of sister cells, which was linked to cell decision after stimulation with death-receptor ligands or conventional chemotherapy. The underlying hypothesis to our approach and others, is that sister cells share the same regulatory network topology, and response heterogeneity should arise mainly from variability in the initial, or baseline, amounts of proteins. To materialize this hypothesis and expand its supporting evidence with the dynamic mechanisms causing cell response heterogeneity, we present a modeling approach utilizing single-cell dynamic data to harness the information contained in each cell of an isogenic cell population. As a whole, this information bears the total capacity of one single cell to produce the required dynamics for a binary cell decision. For a proof-of-principle, we constructed a mass-action mathematical model of the apoptosis receptor reactions (ARRM, Fig. 2), calibrated to single-cell experimental data in order to analyze heterogeneity-driving mechanisms that determine cellular response.

### A positive feedback loop sufficiently amplifies small variations in receptor complex proteins to reproduce live-cell decision observed experimentally

In this work, we identify the heterogeneity-driving mechanisms by ranking the model parameters differences, when fitted to experimental trajectories of resistant and sensitive cell in the same clonal populations under drug treatment. Based on the differences in a group of five parameters (over 32), we tested the hypothesis that the corresponding reactions have an additional regulation by one of the essential proteins, so as to better fine-tune the reaction rate to the current cell’s state and improve its response. This extra regulation step was modeled as a positive feedback loop which was introduced in different possible network configurations. Three of these lead to qualitative changes in the system dynamics due to the emergence of a new stable steady state at the origin and a nearby saddle point, in addition to the expected steady state (Fig. 4). Our results show that, instead of reflecting a bi-modal behavior, both resistant and sensitive phenotypes correspond to the same high caspase-8 steady state. A saddle node has the property that trajectories slowly approach it from a given direction and quickly recede from it along a transversal direction. So, in practice, the saddle node is not directly responsible for phenotype separation but rather generates a large range of dynamics features from a relatively small neighborhood of initial conditions (Fig. 4), thus providing a robust basis for cell response heterogeneity. Consequently, we show that the separation into death or survival phenotypes does not emerge from the model at this stage, but rather from an appropriate combination of initial conditions and TRAIL dose.

### The role of initiator caspases in the positive feedback loop

Our analysis shows that an effective feedback loop between early signaling factors of the extrinsic apoptosis pathway enables cell response heterogeneity, leading us to propose that a sequence of regulatory steps involving only initiator caspases can account for the cell-to-cell variability allowing cell decision. Two hypotheses can be put forward as molecular bases for the positive feedback loop. The first hypothesis is based on data describing the formation of a chain of caspases-8 at the DISC^20,21^. The successive binding of several molecules of the same type can be modeled by a sigmoidal function, of the form used to represent our feedback (see Eq. (1)). The fast recruitment of caspase-8 molecules to form a chain at the DISC can accelerate pC8 binding to receptors and further C8 activation.

As a second hypothesis, we propose a combined effect between caspase-8 and caspase-10 to regulate the binding of pro-caspase-8 to DISC. Indeed, a recent study in HeLa cells has identified new roles for caspase-10, a close homolog of caspase-8^24^. Caspase-10 and cFLIP appear to have similar roles in rendering cells more sensitive, since knockdown of caspase-10 enhances cell death. But the experiments also suggest a cooperative and hierarchical binding of caspase-8 and cFLIP to reveal a similar cooperative binding effect between caspase-8 and caspase-10, since binding of caspase-10 is ineffective in the absence of caspase-8. To further test this relationship between caspases-8 and -10, we developed a more detailed model in^45^, where caspase-10 acts as an intermediary regulatory protein and sets the threshold for the maximal slope of caspase-8 activation.

The consideration of a positive feedback loop in the extrinsic apoptosis pathway is not new^25,26,31,46^, and our analysis supports this hypothesis by showing that several forms of feedback from caspase-8 to upstream reactions may explain cell response (see Table 1). However, three new important contributions arise from our results: first, the feedback amplification happens rather early (less than an hour) upon pathway activation; second, among the main proteins in the pathway (death receptors, FLIP, pC8, C8) only caspase 8 is able to amplify cell response through feedback; and, finally, the molecular mechanism behind the effective feedback loop are likely to involve only initiator caspases.

### Universal cell profiles generate heterogeneity

The second level of our approach identifies a family of model configurations with high capacity in producing heterogeneous responses from perturbations in initial conditions. These model configurations specifically, can then reproduce the variability observed in the experimental data when compared to the original model structure (comparing ARRM and ARRM+Feedback for the same cell (Fig. 5, right panel). Cells with a prototypical behavior near the population average, named *universal cells*, exhibit a remarkable capacity for modulating their responses according to the initial amounts of essential proteins in ARRM+Feedback (Supplementary Fig. 3), in contrast to the original model (Supplementary Fig. 2). This modulation capacity given by the extra regulatory reaction (positive feedback loop, $k_{23}(C8)$), effectively adjusts neighboring rates and amplifies small initial differences in protein expression. This flexibility allows the model to accurately represent the responses of cells to different amounts of TRAIL, calibrating the initial conditions of only four main proteins with a fixed parameters set, for three doses of TRAIL (Fig. 6).

This experimental dependence of the maximal slopes of caspase-8 activity trajectories on initial conditions^7^, is confirmed by model simulations, where randomly generated initial conditions exhibit the same distributions of initial conditions and slopes (compare middle and bottom rows of Fig. 6). The experimental and model results also agree on the different distributions obtained for sensitive or resistant cells, substantiating the idea that variability in early response dynamics between cells emerge entirely from heterogeneity in the initial amounts of molecules, as would be expected in a clonal cell population. With model calibration to single-cell data from an experimental dataset of increasing death-ligand doses, the observed saddle node on the system dynamics bring forth an interesting outcome to the fractional killing observed in cancer pharmacology. Our work suggests that only a specific molecular context (or cell state), described here as specific combination of initial amounts of proteins, will eventually yield a sensitive response to increasing TRAIL doses at the time of treatment. The challenge remains to favor the appropriate initial conditions through co-treatment strategies or cell profile selection, and understand their link to the death-ligand dose.

Here we present an elemental approach to fitting single-cell dynamic data and derive key cell dynamics driving the response heterogeneity that enables drug evasion. Our proof-of-principle study of receptor-mediated apoptosis, show that the resulting models can serve predicting the response of a given cell state to a given drug dose, explaining the origin of the observed fractional killing. Since isogenic cell population naturally harbor cells with switching cell states having differential drug sensitivity^9^, our approach could impact cancer therapeutic development, in revealing the heterogeneity-driving reactions that can be used as co-treatment targets or screening criteria for new therapeutics^47^.

## Methods

### Model calibration

#### Estimating parameters only

In a first step, all reaction constants, as well as synthesis and degradation rates were estimated for each cell separately. To fit each single cell trajectory to the model, we used a standard optimization procedure to minimize the following cost function:

4 $$\begin{aligned} J_P = \sum _{i=1}^{T_Z}\ ( {\widehat{cL}}(i)- cL(i;P) )^2 \end{aligned}$$

where *P* represents the vector of 32 parameters, ${\widehat{cL}}(i)$ represents the observation at instant *i* and *cL*(*i*; *P*) represents the model estimate at the same instant, for the set of parameters *P*.

For each cell, the cost $J_P$ was minimized with respect to *P*, using *fminsearch* in Matlab. The initial guess, $P_0$, for the parameter set was based on values from the literature and was the same for every cell (parameters $k_1$–$k_6$ from^48^, degradation and synthesis from^34^ and all others from^15^). To maintain a reasonable level of comparison between all cells, the initial conditions for solving the differential equations were fixed at the values obtained from the experimental conditions, equal for all cells, and not treated as parameters. The initial conditions are given in terms of numbers of molecules, as follows:

5 $$\begin{aligned} R_0=31400,\ \ F_0=9800,\ \ pC_0=1.47\times 10^5,\ \ B_0=1.9608\times 10^6,\ \ L_0=3.9216\times 10^6, \end{aligned}$$

with TRAIL at $T_0=1500$ (corresponding to the second group of cells, with 50 ng/ml), and all other complexes were assumed to be 0.

#### Estimating initial conditions only

An analogous optimization problem was applied, but now the cost is over the initial conditions of the four main proteins:

6 $$\begin{aligned} J_{IC} = \sum _{i=1}^{T_Z}\ ( {\widehat{cL}}(i)- cL(i;IC) )^2 \end{aligned}$$

where $IC(R(0),FLIP(0),pC8(0),Bid(0))'$ represents the vector of 4 initial conditions, ${\widehat{cL}}(i)$ represents the observation at instant *i* and *cL*(*i*; *IC*) represents the model estimate at the same instant, for the initial conditions *IC*. In this part, the model’s parameters were fixed at those estimated for ARRM+Feedback $k_{23}(C8)$ (see Supplementary Table 1). The initial guesses for this optimization problem were the same as before $IC_0=(R_0,FLIP_0,pC8_0,Bid_0)'$. Depending on the cell’s group, TRAIL was set at $T_0=750$, $T_0=1500$, or $T_0=3000$.

### Parameter distributions

The parameters $k_i$ of ARRM (Supplementary Table 1) are further labeled as

7 $$\begin{aligned} k_{i,Z}^j, \ \ \ i\in I_P=\{1,\ldots ,76\} \ \ Z\in \{sens,res\},\ \ j=1,\ldots ,414, \end{aligned}$$

where *i* represents the parameter (note that, due to parameter simplification, the cardinality of $I_P$ is only 32), *Z* represents the population, and *j* represents cell number (the superscript *j* will be omitted when there is no ambiguity, to ease notation).

The 32 parameters may greatly vary in their orders of magnitude. To more suitably compare the two subpopulations, we work with the logarithms of the parameters, computing parameter averages as follows:

8 $$\begin{aligned} {{\bar{k}}}_{i,sens}= \frac{1}{N_{sens}}\sum _{j=1}^{N_{sens}} \log _{10} k_{i,sens}^j,\ \ {{\bar{k}}}_{i,res}= \frac{1}{N_{res}}\sum _{j=1}^{N_{res}} \log _{10} k_{i,res}^j,\ \ \end{aligned}$$

where $$N_{res}=114$$ and $$N_{sens}=300$$.

### 2D-model analysis

In the absence of any feedback loops, the nullcline $d\textit{T:R}/dt=0$ is a straight line at the (only) concentration ${\overline{\textit{T:R}}}$ which satisfies both the second order polynomial $k_{on} (R_0-\textit{T:R}) (T_0-\textit{T:R}) -k_{off}\textit{T:R}=0$ and the mass conservation laws, given by:

9 $$\begin{aligned} {\overline{\textit{T:R}}}=\frac{1}{2}\frac{k_{off}}{k_{on}}+\frac{1}{2}(T_0+R_0)- \frac{1}{2}\sqrt{\left( \frac{k_{off}}{k_{on}}+(T_0+R_0)\right) ^2-4T_0R_0} \end{aligned}$$

The $dC8/dt=0$ nullcline is given by all pairs $(\textit{T:R},C8)$ satisfying $C8=(k_{act}/k_{def})\textit{T:R}$. Both nullclines are illustrated in Fig. 4a, with $d\textit{T:R}/dt=0$ represented in black and $dC8/dt=0$ in red. The two nullclines intersect exactly once, hence system (2) has a single steady state at $({{\overline{\textit{T:R}}}},C8_F)$ with $C8_F=\frac{k_{act}}{k_{deg}}{{{\overline{\textit{T:R}}}}}$.

The nullclines divide the phase plane into different regions (four in Fig. 4a) where the vector fields have the same signs and define the dynamical behavior of the system. The feedback function modulates one of the nullclines to possibly introduce new intersection points, thus modifying the landscape of the phase plane and possibly affecting the dynamical behavior of the system.

Consider first the case of positive feedback from *C*8 through $k_{on}$, $K_{on}(C8)$. In this case, the red nullcline remains unchanged (since Eq. (3) for C8 remains unchanged), while the black nullcline now depends on C8: $k_{on}$ is multiplied by the expression $C8^2/(C8^2+k_{fb}^2)$ so that ${\overline{\textit{T:R}}}$ is no longer constant but is deformed to look as in Fig. 4e. In addition to the high steady state $C8_F$, the two nullclines now intersect at the origin and at an intermediate point, which turns out to be a *saddle node* (see below).

Consider next the case of feedback from *C*8 into $k_{act}$, $K_{act}(C8)$. In this case, the black nullcline doesn’t change and remains a vertical line at ${\overline{\textit{T:R}}}$, but the red nullcline is now determined by the expression:

10 $$\begin{aligned} k_{act}\frac{C8^2}{C8^2+k_{fb}^2} \textit{T:R}- k_{deg} C8 = 0 \ \ \Leftrightarrow \ \ \left( C8\equiv 0 \ \text{ or } \ \textit{T:R}=\frac{k_{deg}}{k_{act}}\frac{C8^2+k_{fb}^2}{C8} \right) . \end{aligned}$$

This configuration introduces a nonlinear dependence of *C*8 on $\textit{T:R}$ as well as an extra branch in the red nullcline, since $C8(t)\equiv 0$ becomes a solution of Eq. (3). Similarly to the case $K_{on}(C8)$, the two nullclines intersect at three points, as illustrated in Fig. 4c: the high steady state characterized by $C8_F$, a low steady state $({\overline{\textit{T:R}}},0)$ with no active caspase 8, and an intermediate saddle node.

A third example is feedback through the degradation rate, $K_{deg}(C8)$. In this case, the black nullcline is unchanged, a vertical line at ${\overline{\textit{T:R}}}$. But, to obtain a positive feedback through degradation (that is, *C*8 eventually contributes to increase its own concentration), we need to multiply $k_{deg}$ by a decreasing function of *C*8, as follows:

11 $$\begin{aligned} k_{act}\textit{T:R}- k_{deg} \frac{k_{fb}^2}{C8^2+k_{fb}^2} C8 = 0 \ \ \Leftrightarrow \ \ \textit{T:R}=\frac{k_{deg}}{k_{act}}\frac{k_{fb}^2\,C8 }{C8^2+k_{fb}^2}. \end{aligned}$$

The new degradation term $k_{deg}{k_{fb}^2\,C8}/(C8^2+k_{fb}^2)$ is approximately linear for $C8<k_{fb}$, but has a constant limit and tends to zero for large *C*8, implying that *C*8(*t*) grows infinitely large for initial conditions satisfying $C8\gg k_{fb}$. The corresponding red nullcline has the form shown in Fig. 4d and intersects the line ${\overline{\textit{T:R}}}$ at two points, once at the expected high $C8_F$ state and unstable saddle node.

Finally, notice that positive feedback from C8 through $k_{off}$ has an analogous effect on the $\textit{T:R}$ nullcline as the feedback $K_{on}(C8)$. By “positive feedback” we mean that *C*8 eventually contributes to increase its own concentration. Therefore, because the term $k_{off}$ is negative, this parameter must be multiplied by a decreasing function of *C*8, as in the $K_{deg}(C8)$ case. From the dependence of the expression ${\overline{\textit{T:R}}}$ on $k_{off}/k_{on}$, we conclude that the network with $K_{off}(C8)$ behaves similarly to the network with $K_{on}(C8)$.

Among the feedback variables tested (*C*8, *R* and 1/*C*8), only *C*8 is able to generate the desired dynamics.

### Evaluation of heterogeneous response capacity for ARRM+Feedback

To characterize the capacity of each feedback configuration to reproduce cell response heterogeneity, we proceed as follows:

(i) Pick a pair $(X,k_r)$ out of $\{C8,pC8,R,F\}$ and $\{k_5,k_{11},k_{18},k_{23},k_{35},k_{70},k_{72}\}$;
(ii) Set the new reaction $K_r$ to the form given in Eq. (1);
(iii) Perform new single cell data parameter estimation for the corresponding ARRM + Feedback configuration;
(iv) For each fitted cell-model, compute the range of slopes induced by a 20% variation in initial conditions;
(v) Identify cell profiles with the capacity to generate a highly heterogeneous response as those leading to a sufficiently wide interval of slopes:
  12 $$\begin{aligned} \Delta S_i=\frac{\max (s_{itest})-\min (s_{itest})}{s_{exp}}\ge 1.5 \end{aligned}$$
  where $s_{exp}$ is the experimental slope and $s_{itest}$ is the slope response observed for a randomly generated set of initial conditions ($j=1,\ldots ,300$);
(vi) For each configuration, count the number of cells satisfying inequality (12), reported in Table 1.

## Supplementary Information

Supplementary Information.
