# Supplementary material for: Two-level modeling approach to identify the regulatory dynamics capturing drug response heterogeneity in single-cells
Source: Sci Rep. 2021 Oct 21;11:20809. doi: 10.1038/s41598-021-99943-0 (PMC8531316; doi:10.1038/s41598-021-99943-0)
Supplement: Supplementary file 1 — Supplementary Information. [file 41598_2021_99943_MOESM1_ESM.pdf]

Supplementary Information

Two-level modeling approach to identify the regulatory dynamics capturing drug response heterogeneity in single-cells

Madalena Chaves[1], Luis C. Gomes-Pereira[1,2], and Jérémie Roux[2,*]

[1]Université Côte d'Azur, Inria, INRAE, CNRS, Sorbonne Université, Biocore team, Sophia Antipolis, France
[2]Université Côte d'Azur, CNRS UMR 7284, Inserm U 1081, Institut de Recherche sur le Cancer et le Vieillissement de Nice, Centre Antoine Lacassagne, 06107 Nice, France
[*]Corresponding author. Email: jeremie.roux@univ-cotedazur.fr

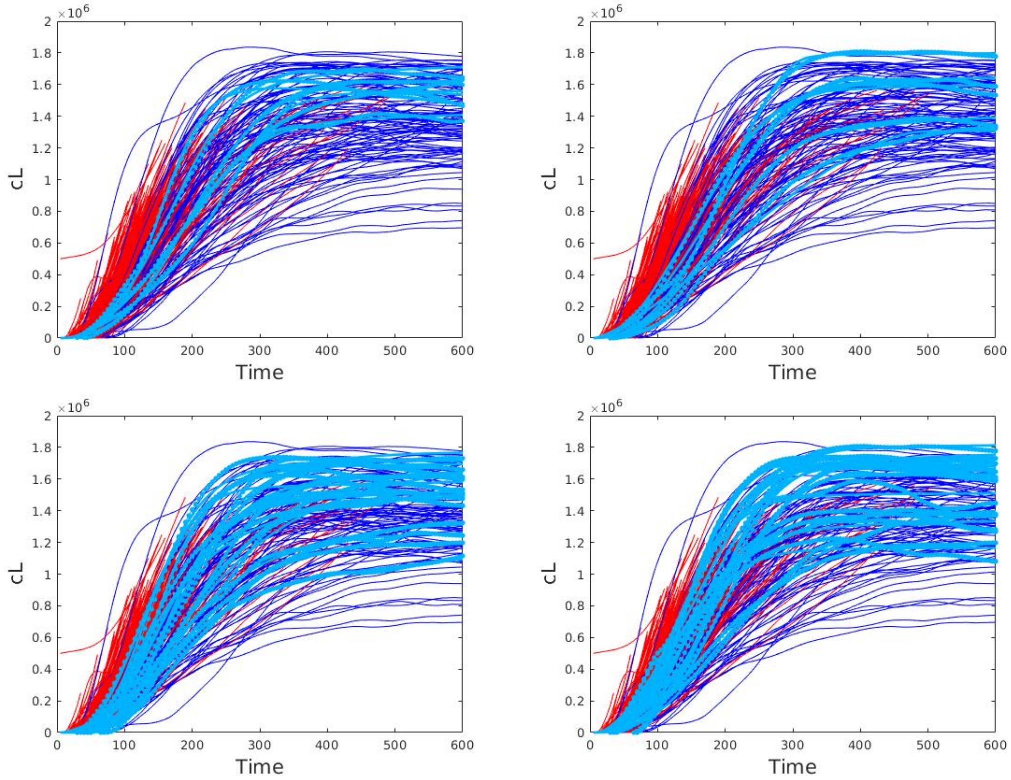

Supplementary Figure 1: All cells with universal profiles detected for ARRM with feedback from $C8$ into k_5 (top left), k_{72} (top right), k_{35} (bottom left), and k_{23} (bottom right).

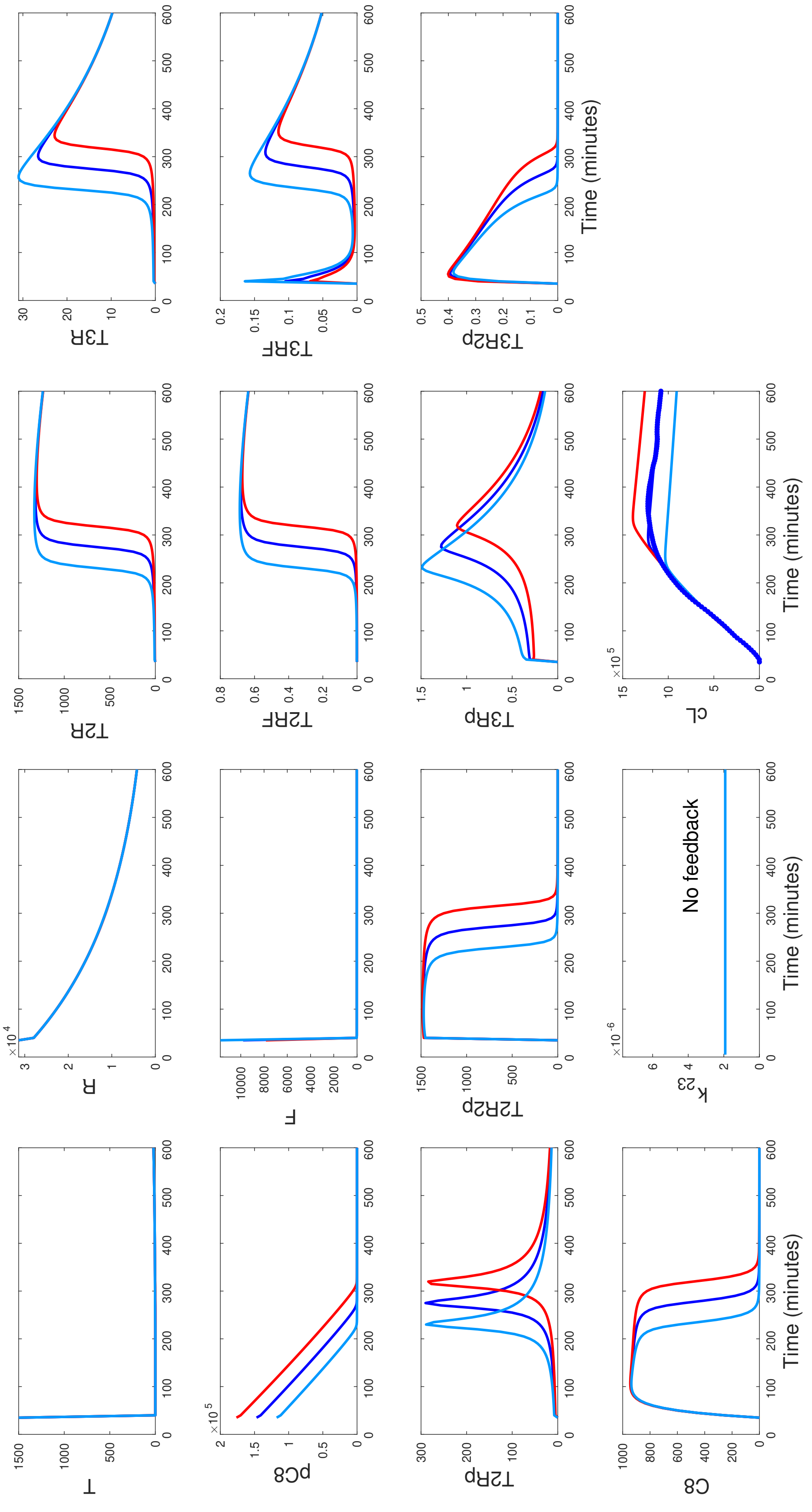

Supplementary Figure 2: Dynamics for cell 271, ARRM with no feedback. Three different initial conditions: nominal ($pC8_0$,$FLIP_0$) (blue), (1.2$pC8_0$,0.8$FLIP_0$) (red), (0.8$pC8_0$,1.2$FLIP_0$) (light blue).

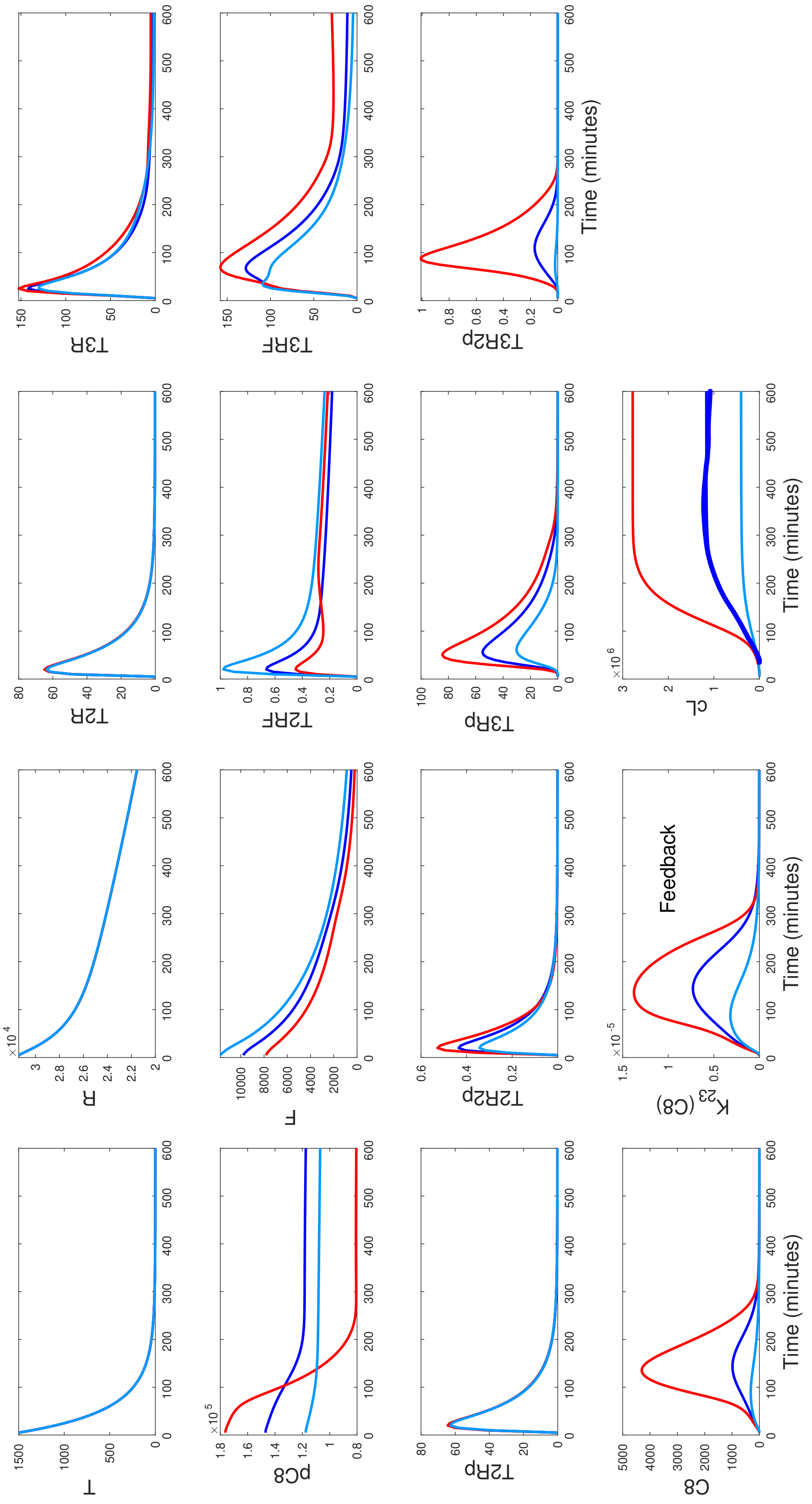

Supplementary Figure 3: Dynamics for cell 271, ARRM with $K_{23}(C8)$ feedback. Three different initial conditions: nominal ($pC8_0$,$FLIP_0$) (blue), ($1.2pC8_0$,$0.8FLIP_0$) (red), ($0.8pC8_0$,$1.2FLIP_0$) (light blue).

Supplementary Table 1: Table of reactions and corresponding parameters, as described by ARRM. The third and fourth columns list the parameter values obtained for cell#271, for ARRM and ARRM+Feedback with $K_{23}(C8)$.

Reaction	Parameter	Cell 271	
		ARRM	ARRM +Feedback
Ligand-receptor complexes			
T +R → T:R	k1 ($\#^{-1}s^{-1}$)	1.2343×10^{-4}	6.6999×10^{-7}
T:R → T+R	k2 (s^{-1})	0.0754	0.0045
T:R +R → T:2R	k3 ($\#^{-1}s^{-1}$)	0.0142	7.1425×10^{-4}
T:2R → T:R+R	k4 (s^{-1})	6.9896	0.0560
T:2R +R → T:3R	k5 ($\#^{-1}s^{-1}$)	2.0065×10^{-5}	1.1673×10^{5}
T:3R → T:2R+R	k6 (s^{-1})	10.7493	0.0080
Ligand-inhibitor complexes			
T:R +F → T:R:F	k9 ($\#^{-1}s^{-1}$)	9.9279×10^{-4}	2.8003×10^{-7}
T:R:F → T:R+F	k10 (s^{-1})	0.0328	0.1442
T:2R +F → T:2R:F	k11 ($\#^{-1}s^{-1}$)	2.5286×10^{-4}	8.8298×10^{-7}
T:2R:F → T:2R+F	k12 (s^{-1})	9.3523	7.8475×10^{-4}
T:2R:F +F → T:2R:2F	k11 ($\#^{-1}s^{-1}$)	2.5286×10^{-4}	8.8298×10^{-7}
T:2R:2F → T:2R:F+F	k12 (s^{-1})	9.3523	7.8475×10^{-4}
T:3R +F → T:3R:F	k17 ($\#^{-1}s^{-1}$)	6.3154×10^{-5}	1.2829×10^{-5}
T:3R:F → T:3R+F	k18 (s^{-1})	0.2394	1.1047×10^{-4}
T:3R:F +F → T:3R:2F	k17 ($\#^{-1}s^{-1}$)	6.3154×10^{-5}	1.2829×10^{-5}
T:3R:2F → T:3R:F+F	k18 (s^{-1})	0.2394	1.1047×10^{-4}
T:3R:2F +F → T:3R:3F	k17 ($\#^{-1}s^{-1}$)	6.3154×10^{-5}	1.2829×10^{-5}
T:3R:3F → T:3R:2F+F	k18 (s^{-1})	0.2394	1.1047×10^{-4}
Ligand- pro-caspase complexes			
T:2R +pC8 → T:2R:pC8	k35 ($\#^{-1}s^{-1}$)	3.4391×10^{-4}	5.6585×10^{-6}
T:2R:pC8 → T:2R+pC8	k36 (s^{-1})	0.1898	0.0063
T:2R:pC8+pC8 → T:2R:2pC8	k35 ($\#^{-1}s^{-1}$)	3.219×10^{-6}	1.113×10^{-6}
T:3R+pC8 → T:3R:pC8	k23 ($\#^{-1}s^{-1}$)	1.9205×10^{-6}	1.8671×10^{-5}
T:3R:pC8 → T:3R+pC8	k24 (s^{-1})	0.0064	0.0085
T:3R:pC8 +pC8 → T:3R:2pC8	k23 ($\#^{-1}s^{-1}$)	1.9205×10^{-6}	1.8671×10^{-5}
Ligand-inhibitor- pro-caspase complexes			
T:2R:F+pC8 → T:2R:F:pC8	k35 ($\#^{-1}s^{-1}$)	3.4391×10^{-4}	5.6585×10^{-6}
T:2R:F:pC8 → T:2R:F+pC8	k36 (s^{-1})	0.1898	0.0063
T:3R:F+pC8 → T:3R:F:pC8	k23 ($\#^{-1}s^{-1}$)	1.9205×10^{-6}	1.8671×10^{-5}
T:3R:F:pC8 → T:3R:F+pC8	k24 (s^{-1})	0.0064	0.0085
T:3R:2F+pC8 → T:3R:F:pC8	k23 ($\#^{-1}s^{-1}$)	1.9205×10^{-6}	1.8671×10^{-5}
T:3R:2F:pC8 → T:3R:F+pC8	k24 s^{-1})	0.0064	0.0085
T:3R:F:pC8+pC8 → T:3R:F:2pC8	k23 ($\#^{-1}s^{-1}$)	1.9205×10^{-6}	1.8671×10^{-5}

Reaction	Parameter	Cell 271	
		ARRM	ARRM +Feedback
Activation of caspase 8 and fluorescent protein			
T:2R:2pC8 → C8 +T:2R	k38 (s^{-1})	0.2012	120.6982
T:3R:2pC8 → C8 +T:3R	k38 (s^{-1})	0.2012	120.6982
T:3R:F:2pC8 → C8+T:3R:F	k38 (s^{-1})	0.2012	120.6982
C8+Bid → C8:Bid	k59 ($\#^{-1}s^{-1}$)	1.8951×10^{-6}	2.1810×10^{-6}
C8:Bid → C8+tBid	k60 (s^{-1})	2.9483	3.9524
C8+L → C8:L	k59 ($\#^{-1}s^{-1}$)	1.8951×10^{-6}	2.1810×10^{-6}
C8:L → C8+L	k60 (s^{-1})	2.9483	3.9524
Synthesis and degradation rates			
→ R	k64 ($\#s^{-1}$)	0.1451	0.0202
→ pC8	k65 ($\#s^{-1}$)	0.0136	0.0016
→ F	k66 ($\#s^{-1}$)	26.9915	0.0061
→ Bid	k67 ($\#s^{-1}$)	50.1310	2.7465
T → ·	k68 (s^{-1})	1.8043×10^{-4}	7.0599×10^{-5}
R → ·	k69 (s^{-1})	0.0034	3.7205×10^{-4}
F → ·	k70 (s^{-1})	1.4043	0.0031
pC8 → ·	k71 (s^{-1})	5.2947×10^{-4}	3.6925×10^{-5}
C8 → ·	k72 (s^{-1})	0.3264	0.0827
Bid → ·	k73 (s^{-1})	0.0152	7.6013×10^{-4}
tBid → ·	k74 (s^{-1})	0.0167	2.4216×10^{-5}
cL → ·	k75 (s^{-1})	3.9264×10^{-4}	5.4624×10^{-8}
→ FP	k76 ($\#s^{-1}$)	3.8961	0.1607
